# Prescriber Phenotypes: Variability in Topical Rosacea Treatment Patterns Among United States Dermatologists

**DOI:** 10.3390/jcm13206275

**Published:** 2024-10-21

**Authors:** Andrew Nicholas, Allison Spraul, Alan B. Fleischer

**Affiliations:** Department of Dermatology, University of Cincinnati College of Medicine, Cincinnati, OH 45267, USA; nichoaw@mail.uc.edu (A.N.);

**Keywords:** rosacea, drug utilization, Medicare Part D, dermatology

## Abstract

**Background/Objectives**: Aggregate prescribing behavior for inflammatory lesions of rosacea has been described, but individual physician behavior has not been characterized. This study aims to assess the modern state of topical rosacea drug selection by analyzing prescribing patterns among individual dermatologists. **Methods:** We assessed utilization patterns of four topical papulopustular rosacea agents in 2021 Medicare Part-D data. K-means cluster analysis identified prescriber phenotypes based on the proportion of claims for each drug by physician. **Results**: Cluster analysis identified four prescriber phenotypes for topical rosacea agents, with the majority favoring metronidazole. In each of the other clusters, metronidazole was co-prescribed alongside the primary agent. Significant predictors of phenotype included patient ages, patient risk scores, and a group practice setting. **Conclusions**: The study reveals nonuniform prescribing patterns for topical papulopustular rosacea treatments among U.S. dermatologists. While aggregate data indicate diverse drug utilization, cluster analysis suggests that individual prescribers tend to use a limited selection of agents.

## 1. Introduction

Rosacea is a common chronic inflammatory dermatosis primarily affecting the skin of the central face with persistent erythema, papules and pustules, flushing, telangiectasias, and discomfort [1]. If left untreated, rosacea can result in facial deformity and substantial psychological distress [2]. There is concerning evidence that as few as 18% of Americans with rosacea are appropriately treated [3]. 

Rosacea characterization and therapy recommendations were originally modeled around four subtypes including papulopustular, phymatous, erythematotelangiectatic, and ocular rosacea [4]. However, updated recommendations promote an individualized approach, which acknowledges overlap between subtypes and aims at targeting mixed features with effective drug choices [5,6]. For the initial management of papulopustular lesions, the National Rosacea Society Expert Committee recommends a selection of topical agents including metronidazole, azelaic acid, sodium sulfacetamide/sulfur, and ivermectin [5]. These agents demonstrate varying efficacy in control trials, with few comparative studies for sulfacetamide, meaning differences in prescribing could impact outcomes [7,8]. Prior population-based studies report a mix of topical rosacea agent use among United States (U.S.) prescribers, which has changed over time [9,10]. A transition to individualized therapy recommendations may suggest that drug choices vary between dermatologists, necessitating a more granular approach to characterize prescribing practices [10].

Prescriber phenotyping is a valuable tool for analyzing the breadth of treatment utilization within the toolbox of available therapies [11]. Phenotyping can help elucidate the alignment between real-world drug selection and current treatment guidelines for this common yet undertreated condition. In this study, we utilized multi-dimensional cluster analysis to uncover distinct phenotypes in how dermatologists select topical papulopustular rosacea (PPR) therapies. 

## 2. Materials and Methods

### 2.1. Data

We examined Medicare Provider Utilization and Payment Data: Part D Prescriber Public Use Files from 2021 [12]. These national claims data include drug events for each provider with greater than 10 claims for a given drug. The study population included all U.S. dermatologists who prescribed at least one topical rosacea agent approved by the U.S. Food and Drug Administration (FDA), recommended for papulopustular morphologies. These agents included formulations of metronidazole, azelaic acid, ivermectin, and sulfacetamide [5]. These data do not allow for analysis of the concentration, coadministration, or frequency of application. Medicare claims data do not provide granular diagnosis information; therefore, we excluded FDA-approved oral PPR therapies (doxycycline), and other topical agents prescribed off-label for papulopustular rosacea (topical retinoids), as these drugs are also prescribed for other non-rosacea skin pathologies. Treatments approved for other rosacea phenotypes, including α-adrenergic agonists for persistent erythema, were excluded as they are not substitutes for PPR-specific therapies [5].

### 2.2. Cluster Analysis 

Individual dermatologist prescribing behaviors were characterized by calculating the proportion of a physician’s claims for each drug. This created four dimensions for clustering prescriber behaviors into phenotypic groups. K-means clustering was selected due to its versatility in analyzing data in multiple dimensions [13]. This technique organizes data such that each observation falls within a cluster with the least distance between means. The optimal number of k clusters was determined a priori using aligned box criterion and cubic clustering criterion techniques. 

### 2.3. Covariates

After clustering Part D claims data, mean patient Hierarchical Condition Category (HCC) risk scores and mean patient age were aggregated by cluster. HCC risk scores represent patient complexity based on disease and sociodemographic risk factors [14]. Rural–Urban Commuting Area (RUCA) Codes were also used to determine the percentage of rural vs. urban practice settings [15].

Each physician National Provider Identifier (NPI) number was cross-referenced from the Part D Providers Data to the Centers for Medicare & Medicaid Services (CMS) Doctors and Clinicians National Downloadable File to obtain physician characteristics including gender, medical school graduation year, and group vs. solo practice [16]. Years of post-residency experience were defined by subtracting four years from the medical school graduation year and then taking the difference with 2021. 

Dermatologist NPIs were cross-referenced within the 2021 CMS Medicare Physician & Other Practitioners Data File to distinguish practice settings as hospitals or outpatient centers [17]. The cost of living within each practice state was assessed using a standardized Cost of Living index, which is scored based on six major socioeconomic factors [18]. We also controlled for socioeconomic status by practice locality using Social Deprivation Index (SDI) scores within each practice zip-code. SDIs are a measure of seven characteristics of socioeconomic status, where higher scores correspond to higher inequality [19]. 

### 2.4. Statistics

Kruskal–Wallis tests were selected to identify differences in drug proportions and to detect significant differences in continuous variables between clusters, as they are not sensitive to violations of normality [20]. Multiple pairwise chi-square (*χ*^2^) analyses were conducted to assess variation in categorical variables. Bonferroni corrections were employed to adjust for multiple comparisons at a significance level of *p* < 0.05. Logistic regression was used to assess covariate predictors of prescriber phenotype. The mostly metronidazole cluster was selected as the reference group due to the large number of metronidazole-only prescribers, reflecting a dominant behavior. 

For this regression, the number of years practiced was defined categorically around quartiles. Means were used to binarily categorize patent HCC risk scores and patient ages by cluster. We used median scores to account for skewed distributions when binarily categorizing Cost of Living and Social Deprivation outcomes. Statistical analysis was conducted using SAS 9.4 (SAS Institute, Cary, NC, USA). 

## 3. Results

### 3.1. Prescriber Patterns 

A total of 6893 dermatologists were identified within the 2021 Part D claims data for having prescribed at least one of four topical agents recommended for the treatment of PPR. Of these dermatologists, 98.7% (95% confidence interval (CI) 98.3–98.9) prescribed metronidazole, 9.1% (8.5–9.7) prescribed azelaic acid, 4.1% (3.7–4.6) prescribed ivermectin, and 1.6% (1.3–1.9) prescribed sulfacetamide (Table 1). 

K-means clustering identified four distinct prescriber phenotypes based on drug utilization. Phenotypic clusters were named after the most common drug prescribed: 1. mostly ivermectin, 2. mostly metronidazole, 3. mostly sulfacetamide, and 4. mostly azelaic acid. Post hoc Kruskal–Wallis tests showed significant differences in the proportion of each primary drug between clusters (*p* < 0.05) (Table 2).

### 3.2. Prescriber Phenotypes 

**Figure 1:**
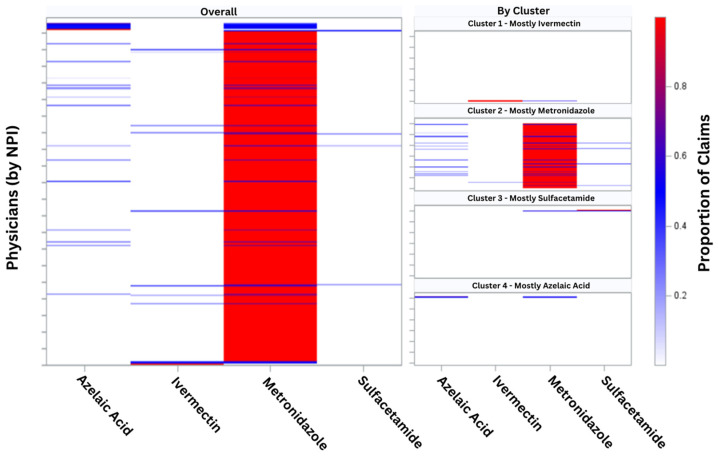
Heat map: proportion of rosacea drugs prescribed by dermatologists. Horizontal lines represent the gradient of prescribing by individual dermatologists across each drug category. National Provider Identifier (NPI).

**Figure 2:**
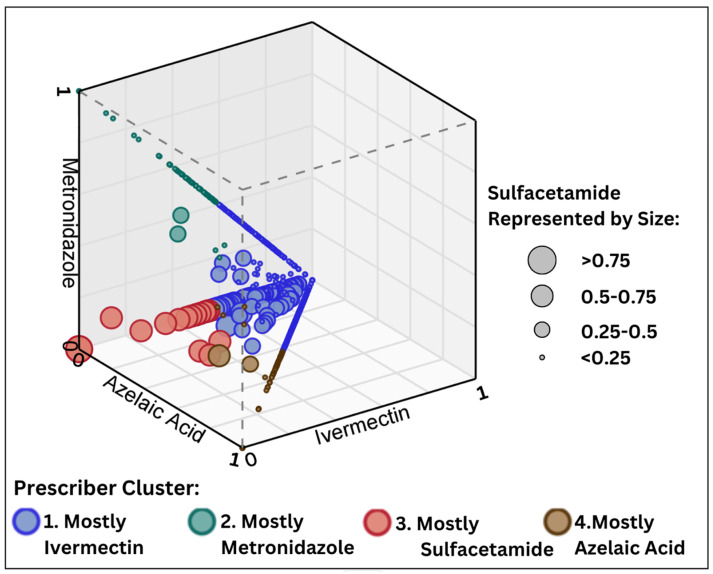
A four-dimensional scatter plot: the proportion of rosacea topicals prescribed by physician cluster. Each physician was plotted in 3 dimensions according to the proportion of metronidazole, ivermectin, and azelaic acid prescribed. The 4th dimension was assigned to the size of plot points, corresponding to sulfacetamide use. Colors were assigned to each cluster to highlight spatial groupings.

The mostly metronidazole cluster was composed of 6642 dermatologists, representing the majority of prescribers. This cohort showed a nearly unilateral preference for topical metronidazole, accounting for 97.4% (97.2–97.6) of claims. Although azelaic acid represented a much smaller proportion of 1.8% (1.6–1.9), a minor tendency toward prescribing this drug was visualized through heat mapping (Figure 1). When compared to the overall prescribing population, this group’s claims mix most closely resembles overall real-world practices. However, the scatter plot representation of each physician’s drug proportions showed that the mostly metronidazole cluster did not have spatial overlap with the other groups (Figure 2). This reinforced that the mostly metronidazole cluster is a group with unique prescribing behaviors, distinct from aggregate data. 

The mostly azelaic acid cluster consisted of 112 dermatologists. Azelaic acid represented 68.7% (64.1–73.2) of their claims and metronidazole was second, at 29.7% (25.2–34.1). Ivermectin and sulfacetamide claims contributed 0.8% (0–1.5) and 0.4% (0–1.0), respectively.

The mostly ivermectin prescriber phenotype captured 111 dermatologists. In this group, 67% (62.4–71.6) of claims were for ivermectin, with a minority of 31.9% (27.5–36.2) attributed to metronidazole. These prescribers had a relatively small proportion of claims for sulfacetamide (0.3% (0–0.8)) and azelaic acid (0.5% (0–10)).

The smallest phenotype comprised 28 dermatologists who prescribed mostly sulfacetamide, which made up 71.3% (61.7–80.9) of their claims. Metronidazole represented 24.5% (15.7–33.2) of claims, which was the lowest among the prescriber groups. While azelaic acid claims were not significantly different among mostly sulfacetamide prescribers, it was prescribed by 14% (1.3–27.3) of these dermatologists, which was greater than the overall mean of 9.1% (8.5–9.8).

### 3.3. Demographic Analysis 

Chi-square tests and Kruskal–Wallis tests showed significant differences in covariate demographic factors between clusters (Table 2). Logistic regression then revealed the strength and direction of these relationships (Table 3). The proportion of solo practitioners was significantly different between the mostly ivermectin and mostly azelaic acid groups relative to the mostly metronidazole cluster (*p* < 0.05). Bivariate logistic regression showed that group practice was a negative predictor for the mostly ivermectin (Odds Ratio (OR) 0.3 (0.2–0.5)), mostly sulfacetamide (OR 0.4 (0.2–0.9)), and mostly azelaic acid (OR 0.5 (0.3–0.8)) clusters when compared to the mostly metronidazole group. In multivariate analysis, this difference remained significant for mostly ivermectin (OR 0.4 (0.2–0.6)) and azelaic acid (OR 0.5 (0.3–0.8)) prescribers.

We observed a significant difference in the proportion of female dermatologists between the mostly ivermectin and mostly metronidazole clusters (*p* < 0.05). Male dermatologists were more likely to be in the mostly ivermectin cluster (OR 1.8 (1.2–2.6)) relative to mostly metronidazole in bivariate analysis; however, this relationship was not significant in multivariate analysis.

Bivariate analysis showed that a greater number of years of practice (1st quartile vs. 4th quartile) was a predictor for being a mostly ivermectin prescriber (OR 1.8 (1.0–3.0)), compared to the mostly metronidazole phenotype. This difference was not significant in multivariate analysis. 

Dermatologists with higher mean patient HCC risk scores (>1.1) were more likely to display a mostly ivermectin vs. mostly metronidazole phenotype in both bivariate (OR 2.1 (1.4–3.1)) and multivariate (OR 2.0 (1.3–3.0)) results. Greater mean patient age was a negative bivariate (OR 0.6 (0.4–0.9)) and multivariate (OR 0.6 (0.4–0.9)) predictor for the mostly ivermectin phenotype relative to mostly metronidazole. Other covariate factors including facility type, urban vs. rural practice, Cost Of Living index, and Social Deprivation Index were not significant predictors of prescriber phenotype.

## 4. Discussion

This cluster analysis reveals four distinct prescriber phenotypes for the use of topical PPR therapies among U.S. dermatologists. This highlights nonuniform treatment practices and a tendency to prescribe a limited range of preferred drugs. Notably, the mostly metronidazole phenotypic group captured the vast majority of dermatologists, revealing a dominant treatment behavior. Despite recommendations for each agent [5], far fewer prescribers used mostly azelaic acid, mostly ivermectin, or mostly sulfacetamide.

Updated rosacea treatment guidelines call for patient-centric care, which may require dermatologists to be flexible in using various topical agents to tailor individualized therapy [5,6]. However, the dominance of metronidazole use may indicate limited patient access to newer treatments like ivermectin and azelaic acid. These drugs vary in efficacy, suggesting possible differences in treatment outcomes between prescribers. Network meta-analysis reports superior efficacy of 1% ivermectin cream to 0.75% metronidazole gel and various formulations of azelaic acid [8]. Among a total of 19 studies, ivermectin users had a greater 12-week treatment success rate and significantly greater reductions in inflammatory lesion counts relative to metronidazole users [8]. In addition to considering efficacy, individualization requires attention to patient perspectives. Studies suggest that high rates of rosacea treatment discontinuation are related to initial treatment intolerance [21,22]. Given that azelaic acid and ivermectin demonstrate similar tolerability to metronidazole, physicians may use shared decision-making to discuss alternatives and guide individualized therapy [7,8]. 

The second and third largest prescriber phenotypes were made up of dermatologists who prescribed mostly azelaic acid and mostly ivermectin, while also using metronidazole. Given evidence from comparative trials demonstrating the superior efficacy of ivermectin and azelaic acid to metronidazole [8,23], these two prescriber groups may be less limiting in offering patients a broader spectrum of efficacious treatment options. 

The smallest and perhaps most unique group of prescribers were those in the mostly sulfacetamide cluster. These dermatologists showed a relatively lower reliance on metronidazole and an above-average use of azelaic acid. This may indicate comfort with a wider range of PPR treatments. Formulations of sulfacetamide/sulfur can be used in combination with other topicals [24,25], which may explain this group’s varied prescribing. However, this group remains small, possibly due to limited comparative efficacy data for sulfacetamide [26].

Dermatologists practicing in a group setting were less likely to be mostly ivermectin or azelaic acid prescribers, relative to the metronidazole group. This contrasts with prior evidence that group practice improved the exchange of information between physicians and encouraged better utilization of care resources [27]. In this case, rather than encouraging diversified prescribing practices, perhaps collaboration in a group setting reinforced tendencies to opt for the predominant use of metronidazole. 

Dermatologists with higher average patient ages were less likely to be ivermectin prescribers. This aligns with prior evidence, suggesting that younger patient age is a predictive factor for ivermectin use, whereas older patients were more likely to be treated with metronidazole [28]. Conversely, dermatologists with patients with higher HCC risk scores, which often correlate with older age and increased comorbidities [29,30], were more inclined to prescribe ivermectin. This could indicate that ivermectin is associated with more complex patterns in topical drug selection within higher-risk patient populations. 

Though we did not evaluate medication cost, we did control for Social Deprivation Index scores and local cost of living metrics, which were not significant predictors of behaviors. It is valuable to note that topical PPR agents were evaluated for cost-effectiveness in a single 2016 study. After consulting 2014 healthcare cost and utilization data, investigators determined that ivermectin 1% cream use contributed to fewer overall out-of-pocket and physician-visit costs relative to metronidazole and azelaic acid formulations [31]. However, these comparisons are dated, offering limited perspective on modern costs, and future studies may benefit from assessing current costs and relationships with prescribing behaviors. Moreover, insurance coverage may dictate prescribing behavior. While coverage patterns vary by insurer, patients and physicians may face greater challenges in achieving coverage for more recently approved therapies, including ivermectin and azelaic acid [32]. 

Our analysis was limited by the lack of disease characterization information within Medicare data. Moreover, the Medicare beneficiary population constrained our analysis of age to those predominantly older than 65 years. Medicare claims data have been valuable for analyzing rosacea therapies prescribed in the U.S. [10]; however, these data do not provide granular information regarding the specific formulations of each topical agent. Topical therapies for rosacea continue to evolve, with the FDA approval of minocycline 1.5% foam in 2020 marking a novel addition for treating papulopustular lesions [33]. However, this agent was not sufficiently represented in 2021 Medicare claims data to be included in this study. Additionally, limited comparative trial data exist for this drug, requiring investigators and clinicians to carefully assess its utility relative to other topical PPR agents [33]. Nonetheless, it presents additional opportunities to offer patients individualized topical options in the modern management of rosacea.

## 5. Conclusions

This study demonstrated that prescriber behaviors among individual dermatologists can vary drastically in selecting topical PPR agents. Cluster analysis showed that most physicians select from a narrow range of preferred topical rosacea therapies, with a vast majority relying upon the use of metronidazole. Although population data showed a mix of drug use, it was not uniform among prescribers. This suggests studying aggregate behaviors may hide the phenotypic differences we saw between physicians. Rosacea can be difficult to treat, as it is associated with high rates of treatment discontinuation, necessitating individualized care. Providing patients with alternative first-line topical agents may help prevent discontinuation or escalation to systemic therapies. We encourage dermatologists to think about their personal prescriber phenotype and their alignment with modern rosacea treatment recommendations. 

## Figures and Tables

**Table 1:** Proportion of dermatologists prescribing each drug by cluster.

	Prescriber Clusters				
	1. Mostly Ivermectin	2. Mostly Metronidazole	3. Mostly Sulfacetamide	4. Mostly Azelaic Acid	Overall
N	111	6642	28	112	6893
Prescribed Ivermectin	100% -	2.5% (2.2–2.9)	3.6% (0–10.4)	4.5% (0.6–8.3)	4.1% (3.7–4.6)
Prescribed Metronidazole	66.7% (57.9–75.4)	100% -	53.6% (35.1–72.0)	62.5% (53.5–71.5)	98.7% (98.3–98.9)
Prescribed Sulfacetamide	1.8% (0–4.3)	1.2% (0.9 1.4)	100% -	1.8% (0–4.2)	1.6% (1.3–1.9)
Prescribed Azelaic Acid	2.7% (0–5.7)	7.7% (7.1–8.3)	14.3% (1.3–27.3)	100% -	9.1% (8.5–9.8)

**Table 2:** Proportion of drug use and practice demographics by cluster.

	Prescriber Clusters				
	1. Mostly Ivermectin	2. Mostly Metronidazole	3. Mostly Sulfacetamide	4. Mostly Azelaic Acid	Overall
Ivermectin Use	67.0% †¶§ (62.4–71.6)	0.5% * (0.4–0.6)	0.7% * (0–2.1)	0.8% * (0–1.5)	1.6% (1.4–1.9)
Metronidazole Use	31.9% † (27.5–36.2)	97.4% *¶§ (97.2–97.6)	24.5% † (15.7–33.2)	29.7% † (25.2–34.1)	94.8% (94.4–95.2)
Sulfacetamide Use	0.3% ¶ (0–0.8)	0.2% ¶ (0.18–0.3)	71.3% *†§ (61.7–80.9)	0.4% ¶ (0–1.0)	0.5% (0.4–0.7)
Azelaic Acid Use	0.5% § (0–10)	1.8% § (1.6–1.9)	3.5% § (0.3–6.8)	68.7% *†¶ (64.1–73.2)	2.8% (2.6–3.1)
% Solo Practitioners	34.2% † (25.4–43.1)	14.3% *§ (13.5–15.2)	28.6% (11.8–45.3)	24.1% † (16.2–32.0)	14.9% (14.0–15.7)
% Outpatient Facilities	98.1% (95.4–100)	95.5% (95.5–96.4)	100% -	99.0% (97.1–100)	96.0% (95.6–96.5)
% Female Physicians	37.4% † (28.2–46.6)	51.3% * (50.1–52.5)	40.7% (22.2–59.3)	54.5% (45.2–63.9)	51.1% (49.9–52.3)
% Urban Practices	94.6% (90.4–98.8)	94.2% (93.7–94.8)	100% -	95.5% (91.7–99.4)	94.3% (93.7–94.8)
Mean Years Practiced	23.6 † (21.1–26.2)	19.3 * (19.0–19.6)	23.1 (18.9–27.3)	21.5 (19.2–23.8)	19.4 (19.1–19.7)
Mean Hierarchical Condition Category Score (by physician)	1.2 †¶§ (1.2– 1.3)	1.1 * (1.1–1.1)	1.0 * (1.0–1.1)	1.0 * (1.0–1.1)	1.1 (1.1–1.1)
Mean Patient Age	73.2 † (72.8–73.7)	73.9 * (73.9–74.0)	73.7 (73.2–74.2)	74.1 (73.7–74.4)	73.9 (73.9–74.0)
Median Social Deprivation Index (by zip code)	38.7 (29.8–47.5)	41.1 (40.1–42.1)	45.5 (25.8–65.2)	36.3 (29.9–42.7)	41.0 (40.1–42.0)
Median Cost of Living Index (by state)	97.8 (94.8–100.8)	101.3 (99.9–102.8)	94.1 (88.8–99.4)	105.8 (96.8–114.8)	101.3 (99.9–102.8)

Kruskal-Wallis tests were used to compare differences between clusters for drug proportions, years of practice, patient risk core, patient age, Social Deprivation Index, and Cost of Living index. *χ*^2^ tests were used to compare solo (vs. group) practice, outpatient (vs. hospital) facility, and urban (vs. rural) practices. * Significantly different from cluster 1 (*p* < 0.05). † Significantly different from cluster 2 (*p* < 0.05). ¶ Significantly different from cluster 3 (*p* < 0.05). § Significantly different from cluster 4 (*p* < 0.05)

**Table 3:** Logistic regression for predictive factors of prescriber phenotype.

	Bivariate Analysis (OR, 95% CI)			Multivariate Analysis (OR, 95% CI)		
Prescriber Clusters:	Mostly IVM vs. MTZ	Mostly SFA vs. MTZ	Mostly AZA vs. MTZ	Mostly IVM vs. MTZ	Mostly SFA vs. MTZ	Mostly AZA vs. MTZ
Group vs. Solo Practice	0.3 ** (0.2–0.5)	0.4 * (0.2–0.9)	0.5 * (0.3–0.8)	0.4 ** (0.2–0.6)	0.4 (0.2–1.1)	0.5 * (0.3–0.8)
Hospital vs. Outpatient Facility	0.5 (0.1–1.8)	0 -	0.2 (0–1.6)	0.5 (0.1–1.9)	0 -	0.3 (0–2.2)
Rural vs. Urban Practice	0.9 (0.4–2)	0 -	0.8 (0.3–1.9)	0.7 (0.3–1.9)	0 -	0.9 (0.4–2.2)
Male vs. Female Dermatologist	1.8 * (1.2–2.6)	1.5 (0.7–3.3)	0.9 (0.6 1.3)	1.3 (0.9–2.1)	2.1 (0.8–5.4)	0.8 (0.5–1.2)
Hierarchical Condition Category Score >1.1 (mean)	2.1 * (1.4–3.1)	0.6 (0.3–1.3)	0.7 (0.5–1.0)	2.0 ** (1.3–3.0)	0.5 (0.2–1.1)	0.8 (0.5–1.2)
Patient Age >73.9 (mean)	0.6 * (0.4–0.9)	0.7 (0.3–1.5)	1.0 (0.7–1.5)	0.6 * (0.4–0.9)	0.5 (0.2–1.1)	0.8 (0.5–1.3)
Cost of Living Index >101.3 (median)	0.9 (0.6–1.3)	0.7 (0.3–1.4)	1.2 (0.8–1.7)	0.8 (0.6–1.2)	0.7 (0.3–1.5)	1.2 (0.8–1.7)
Social Deprivation Index >41.0 (median)	1.0 (0.7–1.5)	1.4 (0.6–3.0)	0.7 (0.5–1.0)	0.8 (0.5–1.2)	1.9 (0.8–4.2)	0.7 (0.4–1.0)
3rd vs. 4th Quartile Years Practiced	0.9 (0.5–1.7)	2.2 (0.6–8.5)	1.1 (0.6–1.9)	1.1 (0.6–2.1)	2.5 (0.5–12.6)	0.9 (0.5–1.8)
2nd vs. 4th Quartile Years Practiced	1.2 (0.7–2.2)	3.2 (0.9–11.8)	1.4 (0.8–2.5)	1.5 (0.8–2.8)	3.5 (0.7–17.4)	1.4 (0.7–2.5)
1st vs. 4th Quartile Years Practiced	1.8 * (1.0–3.0)	2.6 (0.7–10.0)	1.3 (0.7–2.8)	1.6 (0.9–3.2)	2.7 (0.5–14.2)	1.2 (0.6–2.3)

* Statistically significant odds ratio (*p* < 0.05). ** Statistically significant odds ratio (*p* < 0.0001). Clusters: Mostly Ivermectin (IVM); mostly metronidazole (MTZ); mostly sulfacetamide (SFA); mostly azelaic acid (AZA). Year practice quartiles: 1st (≥28 years), 2nd (17.2–28.1 years), 3rd (8.6–17.2 years), 4th (<8.6 years).

## Data Availability

Medicare Part D Prescribers is made publicly accessible by the Centers for Medicare and Medicaid (CMS) at https://data.cms.gov/provider-summary-by-type-of-service/medicare-part-d-prescribers, accessed on 1 September 2023. Medicare Physician & Other Practitioners—by Provider and Service data files are made publicly available by CMS at https://data.cms.gov/provider-summary-by-type-of-service/medicare-physician-other-practitioners/medicare-physician-other-practitioners-by-provider-and-service, accessed on 15 October 2024.
